# Environmental perceptions of global business travel by Swiss companies in the Zurich airport region

**DOI:** 10.12688/f1000research.54862.2

**Published:** 2022-05-05

**Authors:** Ignacio Echeverria Arrondo, Bert Wolfs

**Affiliations:** 1 Student, SBS Swiss Business School, Kloten, Zurich, 8302, Switzerland; 2 Dean, SBS Swiss Business School, Kloten, Zurich, 8302, Switzerland

**Keywords:** Switzerland. Business travel. Covid-19. Environmental awareness. Mobility. Corporate Culture. Leadership.

## Abstract

**Background: **This article presents findings from research conducted before the coronavirus disease 2019 (COVID-19) pandemic on companies located in the Zurich airport region of Switzerland, regarding the needs for global business travel and its impacts.

**Methods:** The study involved a mixed methods approach. Five hypotheses were tested using inferential statistics on data obtained from pre-tested closed questions in a web-based survey. Deeper context was explored through an interview-based case-study conducted at a Swiss pharma company.

**Results:** Supporting alternative hypothesis 3 (Ha(3)), a significant positive relationship was found between travel frequency and business growth, F(1, 100) = 11.31, p = 0.0011. Supporting Ha(4), corporate culture had a significant positive relationship with business travel frequency (F(1, 100) = 15.50, p = 0.0002) and average trip length (F(1, 100) = 6.39, p = 0.01). And thirdly supporting Ha(5), corporate social responsibility had a significant relationship with global business travel (91%). Ho(2) and Ho(3) were accepted. The case study found that smart corporate travel policies and regulations should be instantiated to enhance our environment, which would also benefit employee wellbeing. Travel can be reduced significantly despite being demonstrated that physical co-presence is important for building trust. The case study suggests tools to support the monitoring and management of global business travel by organizations.

**Conclusions:** COVID-19 has impacted travel for business significantly, and future research will be necessary to assess its impact. The article explores the ongoing research in this area, and several relevant implications are proposed for future leaders. The case study found willingness to pay both corporate and individual green taxes, and a deficiency in corporate communication around the environment. Business travel is needed to build trust; however, it can be reduced.

## Introduction

Prior to the 2019 coronavirus pandemic (COVID-19), global air travel had never been more popular. Industry projections expected the industry to serve 20.9 billion air passengers per year by 2040, due to cheaper airfares and increasing flight availability ( Airports Council International, 2018; International Air Transport Association, 2018). Connectivity via flight had never been better. However, this connectivity comes with several downsides for the global community; the best-known of which are greenhouse gas emissions. In Switzerland, carbon dioxide (CO _2_) emissions from air transportation are 4% of the country’s total emissions, of which about a third can be attributed to business travelers ( Flughafen Zürich Ag., 2018).

The proliferation of international business in the era of the pre-COVID-19 global economy generated constant growth in the number of individuals who engaged in long-distance travel for work-related activities ( Gustafson, 2012). Derudder and Witlox (2016) typifies the literature on this growth. In their discussion of mobility and hypermobility, such travel is expected from employees as part of their day to day working lives. It becomes increasingly common as people rise to more senior positions and is supported by a culture which views travel as an expectation, and indeed a privilege to which employees should aspire.

However, others take a more critical stance on the prevalence of travel in business ( Cohen, Hanna, & Gössling, 2018; Gössling, Hanna, Higham, Cohen, & Hopkins, 2019). For many employees, travel is a burden imposed from above, with significant proven health risks and many personal downsides ( DeFrank, Konopaske, & Ivancevich, 2000; Westman & Etzion, 2002). Employees who feel this way face challenges in their lack of enthusiasm for travel, particularly as they climb the corporate ladder.

The study on which this paper is based, conducted prior to the emergence of COVID-19, began when the researcher asked himself: why do we travel so much for business? Is it because we *must* for business-reasons? Or is it mostly because of an established culture of travel? What if we do not travel for business?

As well as the negative impacts on employee wellbeing, the environment provides a pressing rationale for this reconsidering the volume of business travel we undertake. Mr. Antonio Guterres (Secretary-General, United Nations) stated in the COP25 opening ceremony at the Madrid Summit in December 2019, that climate change is now a threat to life across the world. Alongside intergovernmental organizations, climate activists have issued a clarion call for actions such as demanding an end to fossil fuel subsidies and the implementation of restrictions on carbon-emitting activities from worldwide leaders, rather than relying on passive target setting. Their demands, along with the aspirations of the Paris agreement which was formed at UN Climate Change Conference in Paris (COP21), are near-impossible to reconcile with expectations of continued growth in air transport and business travel.

This article reports the findings of research on companies located in the Zurich airport region of Switzerland to illustrate the relationship between environmental awareness and business travel. It also investigates why the subjects of the research travel for business, is it for business growth or is there a broader cultural phenomena divorced from business needs playing a role. Finally, the article asks employees what would happen if corporate social responsibility (CSR) was integrated more widely in business travel policies: would the patterns of business travel change?

The article starts with a survey of the background literature including a section on the Swiss context. The objectives of the study and methods used to investigate them follow. The research was mixed-methods, and details are provided on both the quantitative and qualitative methods utilized. The results section reports results regarding business travel and environmental awareness, business travel and business growth, and corporate culture and social responsibility. The article ends with a discussion, including the implications for future research and suggestions for corporate leaders.

### Background literature

The various explanations of why we travel for business have been the subject of study for several years. Above all, the context is one in which current business leaders and executives have a strong preference for face-to-face meetings over technological alternatives ( Koyen, 2009; Harvard Business Review Analytic Services, 2009). There are two areas in which that preference is to an extent backed up by research: the transfer of knowledge, and trust-building. Both are better accomplished in some circumstances by face-to-face communication, and the ensuing associated travel, than through any of the available virtual alternatives ( Tani, 2005; Welch, Welch, & Worm, 2007). However, the environmental load, and the negative impacts of extensive travel on employee health and retention, are both pressing.

A gap in the literature linking the environment with corporate social responsibility (CSR) was clearly identified as early as 1994: “this concern for the environment serves as a new, unifying theme for the study of international business, which will no doubt continue to deepen and grow in importance” ( Wright & Ricks, 1994, p. 699). The concern is often prompted by large-scale and widely reported disasters, along with responses to governance issues en-vogue at various historical moments. In some areas it has received attention – there are a range of studies on resource use, production-based pollution, and the range of responses firms can take from internal policies to engagement with regulatory bodies.

There are many barriers for companies considering instigating policies to reduce air travel. In some ways, the idea of restricting mobility in any way remains taboo in modern society. That reluctance to consider reducing mobility applies particularly strongly to travel for business, aside from a few scholars including the group who conducted an exploratory study which suggests, among other things, that people consider their professional travel more urgent and valued than their leisure travel ( Gössling *et al.*, 2019). That argument has been made elsewhere, particularly in studies involving academics themselves ( Sahakian *et al.*, 2021). Even among climate change researchers regular international travel is both a norm and considered more important than leisure travel ( Whitmarsh *et al.*, 2020). The taboo around mobility reflects in what researchers choose not to research, too. As one example, there is no study of the “cost of aviation-related pathogen distribution ( Gössling, 2020).

Overall, awareness of the issues corporate travel raises for both employee wellbeing and the environment remains sparse, and further studies are needed to highlight options that might work in particular settings ( Kolk, 2016). This is a particularly important moment to contribute to this discourse because while the COVID-19 pandemic has seen a huge drop-off in air travel, there are indications that many businesses intend to resume travel in the near-term, if they have not already. As has been argued in many fields, recent events may provide a one-off opportunity to reset travel on a more sustainable footing, and to break the hold that travel has over business culture ( Koonin, 2020; Hepburn *et al*., 2020; Kuzemko *et al*., 2020). The current normal remains partially remote, and in some areas, it is allowing equivalent if not superior functionality to in-person communication ( Clopper, Baccei, & Sel, 2020). The potential for an increased use of technology to replace travel has been building for a decade or more ( Julsrud, Denstadli, & Hjorthol, 2014). Following COVID-19 and the necessity of replacing face-to-face communication with technology-mediated forms, that potential may now be realized. This article, the research for which was conducted prior to COVID-19, demonstrates that not all business travel is essential, and will add to the evidence-base that argues a more targeted and specific approach to business travel would benefit employees, the environment, and businesses in the future ( Poom, Orru, & Ahas, 2017). Leaders of the future should de-normalize travel both in how they act and the policies they engage in for their employees.

### Switzerland

Switzerland is a small landlocked country located in central Europe, with a population of 8.5 million people, as of the 2018 census ( Federal Statistical Office, 2018). Switzerland receives almost double the number of travelers that would be anticipated from its expenditure ( Bripi, 2019). This indicates that Swiss companies attract business travelers disproportionately to their size, and this can be applied doubly so in the Zurich area. The study focuses on the Zurich airport region in Switzerland; a population of around 129,329 inhabitants in the following communities: Bassersdorf, Oberglatt, Rümlang, Opfikon-Glattbrugg, Wallisellen, Wangen-Brüttisellen, Dietlikon, Dübendorf, Nürensdorf and Kloten. Zurich Airport (2018) states that in 2017, 31 million passengers used Zurich airport, of which 27% (or 8.37 million passengers) were international business travelers.

How business travel is viewed within a country varies not just with frequency but also with the different perspectives that people bring to bear on it. Switzerland is a country where the people are widely known to be environmentally friendly, as was shown in Sevä and Kulin (2018) which ranked Swiss citizens alongside New Zealanders as the most likely of the 32 countries studied to both demonstrate environmental concern and act in accordance with that concern. Research also demonstrates the consequential value that the Swiss place on their time; for instance, Bates, Bierlaire, Abay, Axhausen, and König (2006) examined how much one hour of traveling was worth to people in different countries within Europe. They found that Swiss citizens place a higher value on their time than any other European citizens do. In Swiss culture, free time and traveling are signs of status – with first class travel and particularly air travel acting as additional signifiers.

Two studies provide insight into how the Swiss view air travel and business travel. Firstly, Bruderer Enzler (2017) provides a short account of air travel for private (non-corporate) purposes in Switzerland. Of interest is the brief examination of “attitudinal predictors”, which analyze behavior on a range of factors based on surveying undertaken in 2007. Of most relevance here was the finding that while those who voted for the Green Party of Switzerland, caused lower emissions overall, they flew nearly as often as the rest of the population. Hinnen, Hille, and Wittmer (2017) found that a significant proportion of passengers might be willing to pay extra for “green” products associated with air travel, such as organic on-board food or carbon offsets.

### Objectives

The main aim of the study was to help the author understand two questions: why do we travel so much for business. Is it because we have to, or because we need to? To answer these, a study was undertaken using both quantitative and qualitative research methods focused on businesses within the Zurich airport area in Switzerland. The study will contribute a detailed case study to the literature, with a greater focus on the intersection between environmental awareness and business travel than previously published work.

## Methods

This study followed a mixed-methods approach, utilizing both a survey and a detailed case study. This approach was chosen in order to provide complementary data on the questions being studied, in the hope that the case study interviewees would elicit explicatory detail that could not be collected quantitatively. This section will outline the main methodological features of the study and give detail as to the characteristics of both the survey respondents and case study. Hypotheses were developed based on secondary literature research before the study was undertaken and are reported in the text below. All study materials can be found as extended data ( Echeverria-Arrondo & Wolfs, 2021).

### Quantitative survey

In total, five hypotheses were tested quantitatively using pre-tested closed questions in a web-based survey, which was collecting data from September to December 2019.

• Ha(1) “As environmental awareness (from the corporate) increases, business travel decreases”.
• Ha(2) “As environmental awareness (from the individual) increases, business travel decreases”.
• Ha(3) “There is a significant positive relationship between global business travel and business growth”.
• Ha(4) “Corporate culture is the predominant reason business travel is undertaken as frequently as it is”.
• Ha(5) “As corporate social responsibilities policies linked to environmental awareness (from the corporate) increases, business travel decreases”.

Information regarding the operationalization of the three major constructs, along with the evaluation of specific hypotheses, can be seen in Table 1. Each construct is listed along with any relevant variables, and the specific questionnaire items that form one. The six columns on the right denote which of the five specified hypotheses are being tested.

**Table 1.** Questionnaire items corresponding to constructs, variables, and hypotheses.

Construct	Variable	Question Text	Question	H1	H2	H3	H4	H5
**Global Business Travel**	**Travel Frequency**	On average, how often do you travel for business?	4			x	x	x
	**Trip Length**	How long are each of your business trips, on average?	5					
**Corporate Culture**	**Business Growth**	Travelling for business increases business growth.	1			x		
		In my experience, business travel has been essential to achieving the results I have.	8					
	**Meeting Culture**	I enjoy travelling for business.	2				x	
		Video/audioconferencing is preferable to a business trip.	6					
		Information Technology (IT) has reduced the use of air travel for business purposes.	7					
**Environmental Awareness**	**Climate Science Knowledge**	I understand the greenhouse effect, its causes, and its consequences.	9	x	x			x
		I understand the greenhouse gas emissions caused by an aircraft.	10					
		I understand the consequences of global warming.	11					
	**Social Responsibility**	I am willing to pay more, as an individual, when purchasing pollution products and services, through “green taxes”.	16			x		
		I am willing to pay (as an individual) a fee to NGO’s such as “myClimate”, when purchasing pollution products and services--business related--through an off-setting carbon footprint compensation.	18					
		I am willing to pay more, as a corporation, when purchasing pollution products and services, through “green taxes”.	17		x			

The questionnaire designed by the researcher was tested for validity and reliability using Qualtrics test survey tool. Pilot testing was undertaken with 10 people in August 2019. The criteria for selection of these 10 people was to hold a role in a company involving traveling, two with native English, and the rest German. They were asked beforehand to check their willingness and availability for participation. They were asked to highlight any unclear questions and to provide overall feedback which was used to improve the final survey. This was especially important as the survey was bilingual, in German and English, with the researcher not a native speaker of either (see Appendices A and B). The participants also timed how long the test took to complete, in order to assess whether it needed to be shorter, or any questions amended. The researcher made some corrections based on the feedback to make the questionnaire clearer avoiding ambiguity and corrected the German language.

There are several hypothetical questions within the set, in which the individual gives their opinion regarding something regarding the business they work at. This offers a limitation of the study, particularly in regard to hypothesis 5. However, the quantitative data and interview discussion should shed light on the underpinning issues. There is also evidence that individuals do, in aggregate, represent the corporation they are working at on issues of preference. For instance, being in a pro-business-travel meeting culture makes it ultimately more likely for an individual to believe they enjoy business travel ( Bentley, Bloomfield, Davidai, & Ferguson, 2016; Mueller, 2010).

The study is based on the region surrounding Zurich airport, in which the latest research available (from 2014) states that there are 118,626 people employed ( Flughafen Zürich Ag., 2014). Informal consultations with local human resources (HR) managers resulted in an estimate that 30 to 40 percent of all jobs within the Zurich Airport region involve global business travel, and thus might fall under consideration for this study. Therefore, the appropriate total population size for this study is 36,000 individuals. Mugenda and Mugenda (2012) posited that where a research population is large, the recommended minimum sample size is 384. This is predicated on the assumption that the population is normally distributed, and the degree of confidence is 95%, at a significance level of five percent.

The study utilized multistage sampling, in which clusters of the overall population are selected and then random samples drawn from each of those clusters. This allows a good balance of cost, convenience and accuracy compared to alternative sampling methodologies ( Gall, Gall & Borg, 2007). In this case, clusters were formed of companies of any sector with which the researcher had a pre-existing relationship. A random sample of their employees was then taken for the survey. Participants were contacted, either by phone or by email, to gain their consent to invite them to take part in the survey.

The survey was sent to target 400 respondents in September 2019. Given normal survey response rates this was unlikely to result in enough responses to meet the 384-sample size; but as a mixed-methods study it was felt to be sufficient for the quantitative arm. The survey was conducted through Qualtrics software, Version 2019 to collect the data and perform quantitative statistical analysis. Google Forms or SurveyMonkey could have been used as open access survey alternatives, paired with Apache OpenOffice for quantitative analysis.

For each question of the survey, responses were coded numerically along a seven-point Likert scale ranging from “very strongly disagree” (coded as -3) to “very strongly agree” (+3), with “neither agree nor disagree” as the neutral option (0). This allowed examination of the correlation between different responses. Then Pearson’s correlation co-efficient was utilized to draw conclusions about the strength of the correlation. Standard deviations provided information on the breadth of opinion. Regression analysis was performed, where warranted, to illustrate the relationship between the variables. Analysis of variance (ANOVA) was used in inferential tests when determining the strength of influence that the independent variables had on the dependent variable.

### Qualitative case study based on in depth interviews

A case study, the interviews for which were carried out between August and September 2019 at a Swiss pharma company in the region provided further in-depth understanding of the reasoning and intentions behind the business travel. Ms. Silvana Micheli, a Swiss citizen native in German, Swiss-German and very high level of the English language, was trained by the researcher to perform the interviews on his behalf to avoid any bias or influencing the interviewees. Ms. Silvana Micheli is a Learning and Development professional in the pharma industry.

In the organization, between 30 and 40 percent of all roles involve traveling. Overall, 15 employees were selected for the case study, a number which was felt to be sufficient for additional detail without risking data saturation. They were identified starting from the nominal description: gender, age, role grade and type of role and department. Only those with a role involving traveling were included. They were intentionally chosen to represent a broad scale of seniority and approached by email for their consent to participate in August 2019. At that time, they were informed of the nature of the study and the topic area, but not the research.

An NDA (non-disclosure agreement) was issued and signed by representatives from the pharma company. The male researcher countersigned it to preserve the anonymity of the company and its employees. The data collected has been stored at SBS Swiss Business School safely and will be kept for five years. After this period, under the terms of the NDA the material including audio recordings of the interviews will be destroyed.

An interview format was implemented with participants and questions were based upon the structure of the survey questionnaire to enable meaningful comparison of results. This gave the interviews a clear structure while allowing the possibility of extended answers and follow up questions as appropriate. Sessions were 45 minutes long, one on one, took place in the offices of the pharma company, and audio was recorded for the purpose of this research study only. The interviewer also took notes, manually writing down the answers to the interview questions. No repeat interviews were necessary. No participant refused to take part. Transcripts were not made available to participants. At the beginning of each interview the interviewer read the consent for audio recording and provided two copies to be signed. The interview audio analysis was part of a broader study than reported here: the answers to the questions were also manually written on the interview questionnaires. The interview transcripts were analyzed using a deductive approach, which was most suitable given the structure of the interviews and the availability of quantitative data for comparison ( Palys & Atchison, 2014). Given the small numbers of interviews this was conducted manually, with no specialist software involved.

### Ethical approval

An ethics declaration form was submitted to and approved by SBS Swiss Business School Human Resources Ethics Committee (approval number: 642). Additionally, a research panel consisting of a supervisor assigned by SBS Swiss Business School and industry specialists, examined the quantitative and qualitative surveys and this research proposal, before giving ethical permission for the study.

## Results

In total, 104 completed questionnaires were received in response to the quantitative study ( Echeverria-Arrondo & Wolfs, 2021). This was a response rate of 26 percent, which given that rates as low as under 10 percent are not uncommon with web surveys, was reasonable. While this sample was sufficient for this mixed-methods study, it is below the 384 required for the quantitative data to be taken as statistically representative of the population being studied. Future researchers may want to replicate this study to a larger sample, and also to take account of the advice in van Mol (2015) and provide repeated digital reminders for potential participants. To prevent lengthening the quantitative survey, which might have further impacted the response rate, demographic data was not collected.

For the case study, four of the 15 participants were female, and 11 were male (26%, 74% male). Their ages ranged from 25 to 65: 25-34: 3 (20%), 35-44: 4 (27%), 45-54: 6 (40%), 55-65: 2 (13%).

### Business travel and environmental awareness

**Figure 1.**
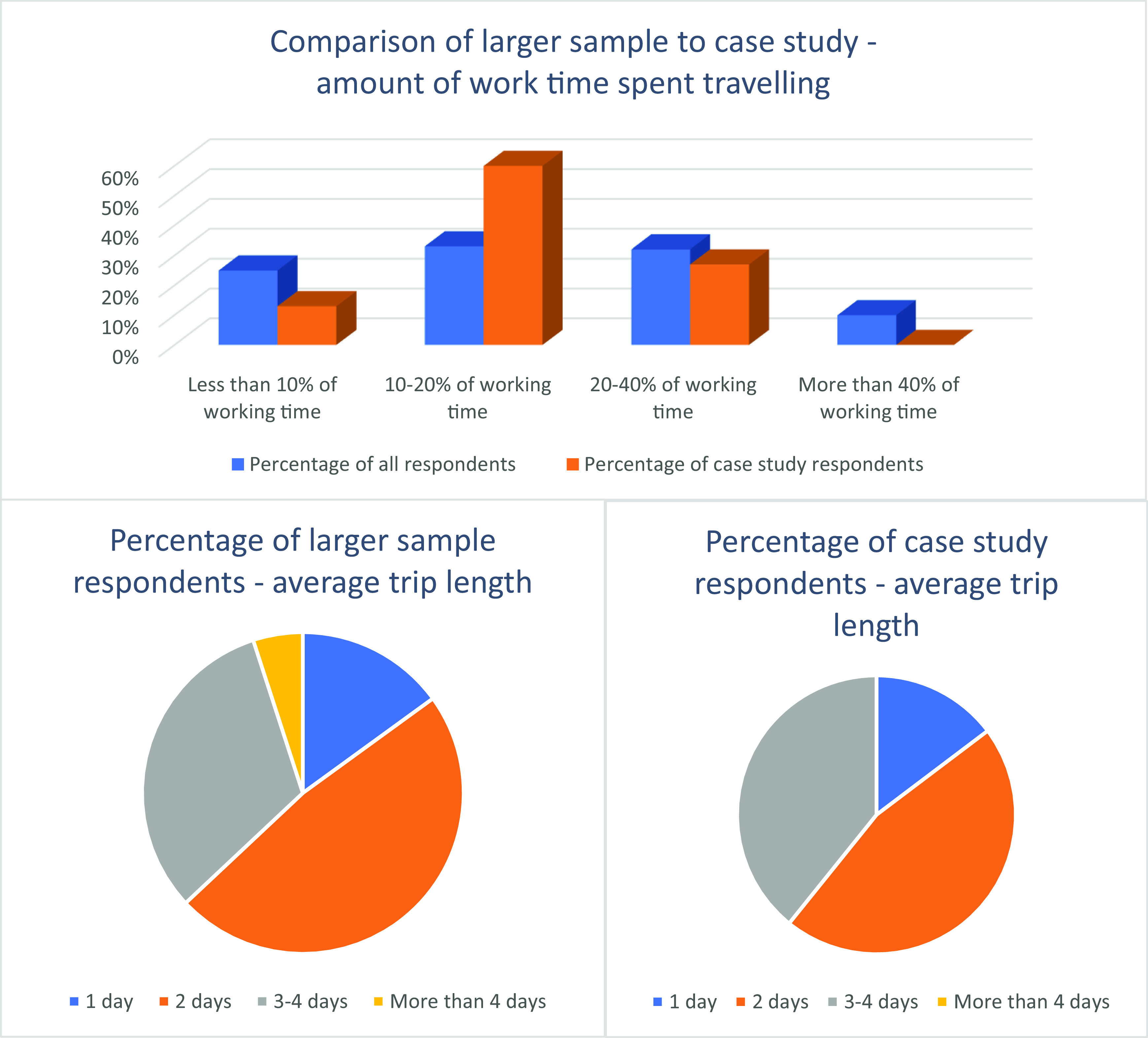
Percentage of work time travelling and trip length.

Travel time and trip length varied markedly among both survey respondents and case study participants, as is shown in Figure 1. A Pearson’s Chi-squared test of independence (with Yates’ continuity correction) between travel frequency and trip length was not significant, χ ^2^(1) = 3.31e-31, *p* = 1. It was therefore acceptable to use them as independent variables for inferential statistics.

Following Ham, Mrčela, and Horvat (2016) this study defined environmental awareness not only as a knowledge of environmental issues, but also a willingness to act on climate science as a corporation *and* as an individual. In business, which tends to be financial remuneration to the global citizenry in some form of green tax or financial offsetting of carbon footprint. As shown in Table 1 above, there were thus two question sets which, taken together, illustrate ‘environmental awareness’.

The first question set regarded climate science knowledge, the correlation matrix for these three questions is displayed in Table 2. The three questions were highly positively correlated with one another, with Pearson R values ranging from 0.75 to 0.89. Since they are so strongly correlated, they are combined into one overall variable called “climate science knowledge” by averaging them together. The LIKERT scale was coded “very strongly disagree” as -3 to “very strongly agree” as +3. Scores on this measure ranged from -0.333 to + 3.00, with a mean of 1.88, and a standard deviation of 0.876.

**Table 2.** Correlation matrix for questions about climate science knowledge.

	Greenhouse effect	Aircraft emissions	Global warming
**Greenhouse effect**	1.00	0.89	0.75
**Aircraft emissions**	0.89	1.00	0.75
**Global warming**	0.75	0.75	1.00

For each of the three statements, the percentage of respondents who endorsed the statement (“understand”, “strongly understand”, “very strongly understand”) was over 95%.

The second question set regarding environmental awareness was regarding social responsibility and willingness to act in regard to the environment. As can be seen in Table 3, the three questions were positively correlated with one another, with Pearson R values ranging from 0.58 to 0.65. In some of the analyses below, a combined variable identified as “social responsibility”, was utilized, created by averaging them together. Scores on this measure ranged from -0.333 to + 3.00, with a mean of 1.88, and a standard deviation of 0.876. The component questions behind this have value alone, too, and are used where appropriate.

**Table 3.** Correlation matrix for questions about social responsibility toward the environment.

	Corporate tax	Individual tax	Individual off-setting
**Corporate tax**	1.00	0.65	0.58
**Individual tax**	0.65	1.00	0.62
**Individual off-setting**	0.58	0.62	1.00

### Ha(1), “as environmental awareness (from the corporate) increases, business travel decreases”

This was not proven from the data collected. There was no significant difference between business travel and willingness to pay corporate taxation. For ‘more’ and ‘less’ frequent travelers, *F*(1, 100) = 0.041, *p* = 0.840. Therefore, the null hypothesis was accepted. No effect was found upon trip length, *F*(1, 100) = 0.33, *p* = 0.86, nor was there any interaction between the two business travel variables, *F*(1, 100) = 1.96, *p* = 0.165.

### Ha(2), “as environmental awareness (from the individual) increases, business travel decreases”

Again, the null hypothesis was accepted here with no statistically significant relationship being found. The analysis did not find a significant difference between more and less frequent travelers, *F*(1, 100) = 0.001, *p* = 0.979. The analysis did not find an effect on trip length, *F*(1, 100) = 0.18, *p* = 0.68, nor any interaction between the two business travel variables, *F*(1, 100) = 0.01, *p* = 0.93.

**Figure 2.**
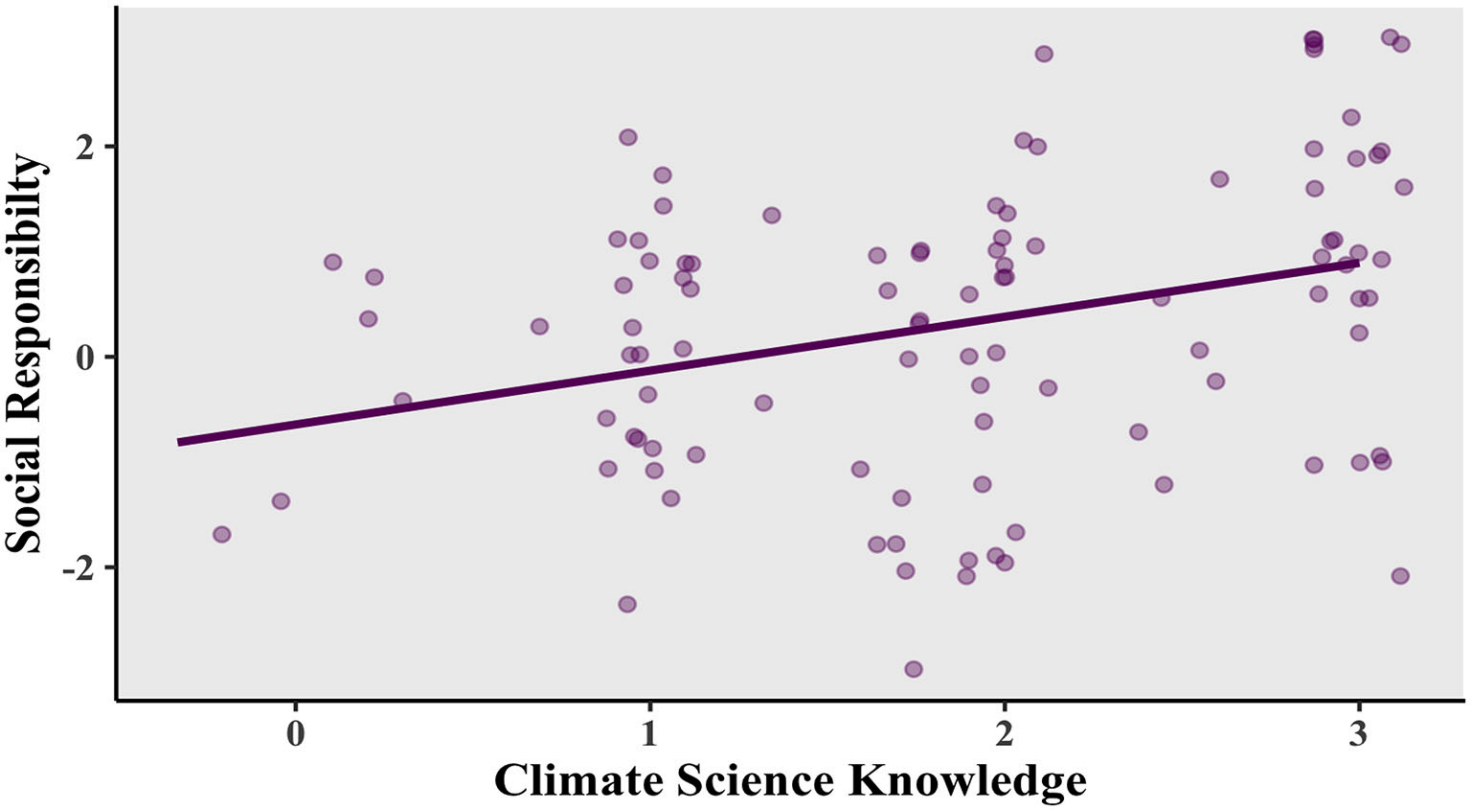
Relationship between social responsibility and climate science knowledge. Scatterplot and Pearson correlation coefficients.

There was a noticeable relationship between the variables making up environmental awareness at the corporate level: “climate science knowledge” and “corporate social responsibility”. As shown in Figure 2, a positive correlation was found, Pearson’s product-moment correlation *R* = 0.309, 95% confidence interval (CI) = 0.124-0.473, *t*(102) = 3.282, *p* = 0.0014.

**Figure 3.**
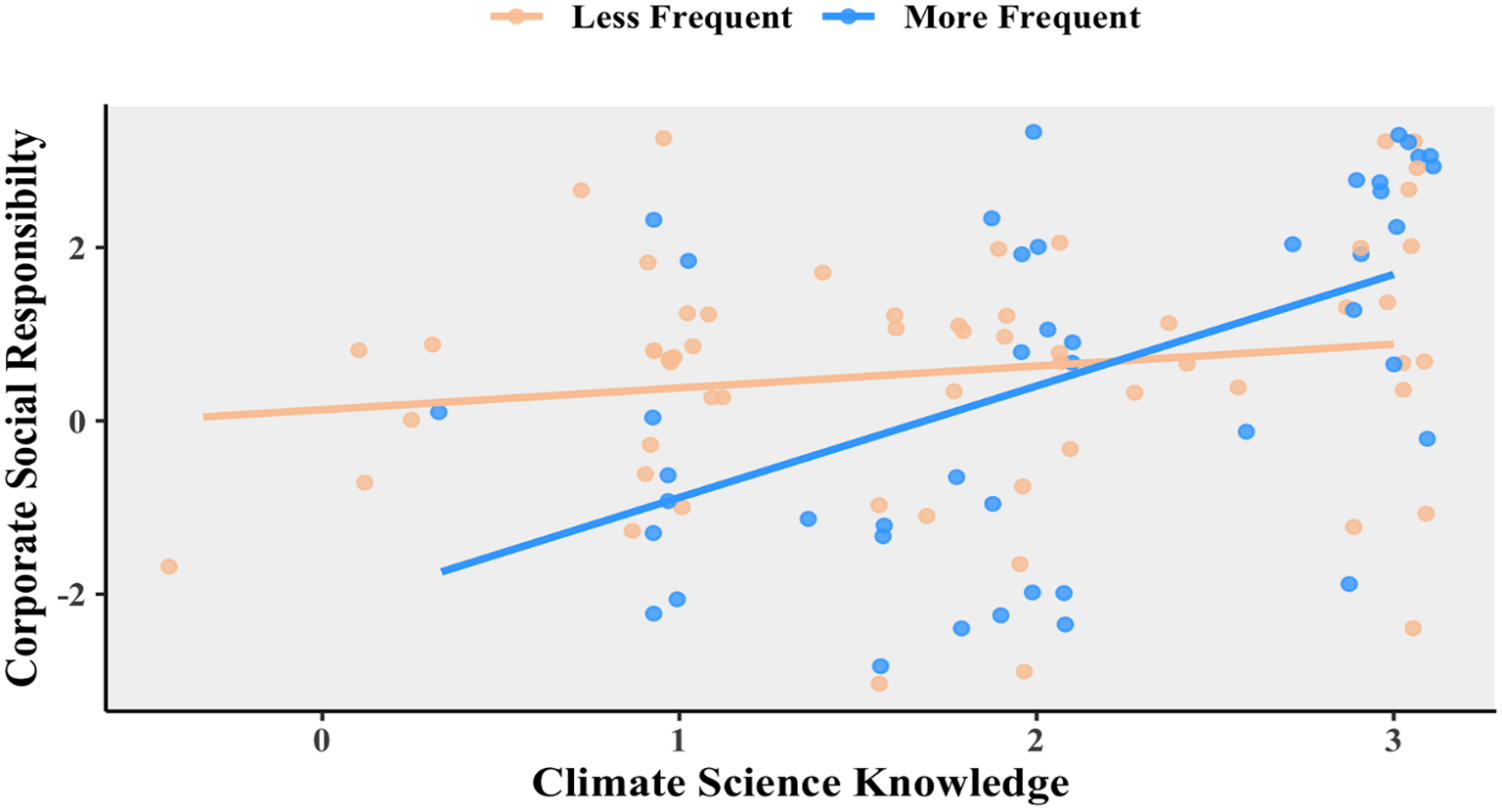
Relationship between corporate social responsibility and climate science knowledge for less frequent (orange) and more frequent (blue) travelers. Scatterplot and Pearson correlation coefficients.

The next question which needed addressing was: is this relationship affected by business travel? To examine this more closely, the respondents were split into more frequent and less frequent travelers to be plotted in that relationship. This can be seen in Figure 3. For those who tend to travel more frequently, “climate science knowledge” and “social responsibility” are positively related, *R* = 0.528, 95% CI = 0.274 – 0.713, *t*(42) = 4.03, *p* = 0.0002. However, there is no such correlation for those who travel less frequently, Pearson’s product-moment correlation *R* = 0.160, 95% CI = −0.097 – 0398, *t*(58) = 1.24, *p* = 0.221.

The analysis combined all the variables that were found related to global business travel, here operationalized as travel frequency. The researcher discovered that elements of “corporate culture” were related to “business travel”: liking business travel; and believing business growth comes from travel. It was also found that an interaction of the elements of “environmental awareness” were related to “business travel”: similarly, “science knowledge” and “corporate social responsibility” are related.

### Business travel and business growth

**Ha(3), “there is a significant positive relationship between global business travel and business growth”.**

As displayed above in Table 1, two questions were asked regarding the relationship between business travel and business growth. To test this hypothesis, the answers were considered on a branching basis, the results from which are displayed in Table 4.

**Table 4.** Business travel and growth question responses.

	Q8 Positive response	Q8 Negative response
**Q1 Positive response**	62	12
**Q1 Negative response**	18	12

A Pearson’s Chi-squared test of independence (with Yates’ continuity correction) was statistically significant, χ ^2^(1) = 5.5283, *p* = 0.01871. Given that dependence, for each respondent “business growth” was calculated by taking the average of the responses from Questions 1 and 8.

If global business travel is undertaken partly because people believe it contributes to the growth of the business at which they work, it would be expected that travel frequency be related to business growth. However, it would not necessarily be expected that the variables would be dependent to the extent that it would predict an average trip length. That is, those who travel more frequently do so because they believe it is integral for their business growth, and that trip length should not be related.

A 2 (trip length: shorter, longer) × 2 (travel frequency: less frequent, more frequent) between-subjects ANOVA was conducted to see whether “business growth” varied across the groups. The data showed that there was a significant difference for travel frequency: indeed, those who travel more frequently did rate travel as more important to their business growth.

**Figure 4.**
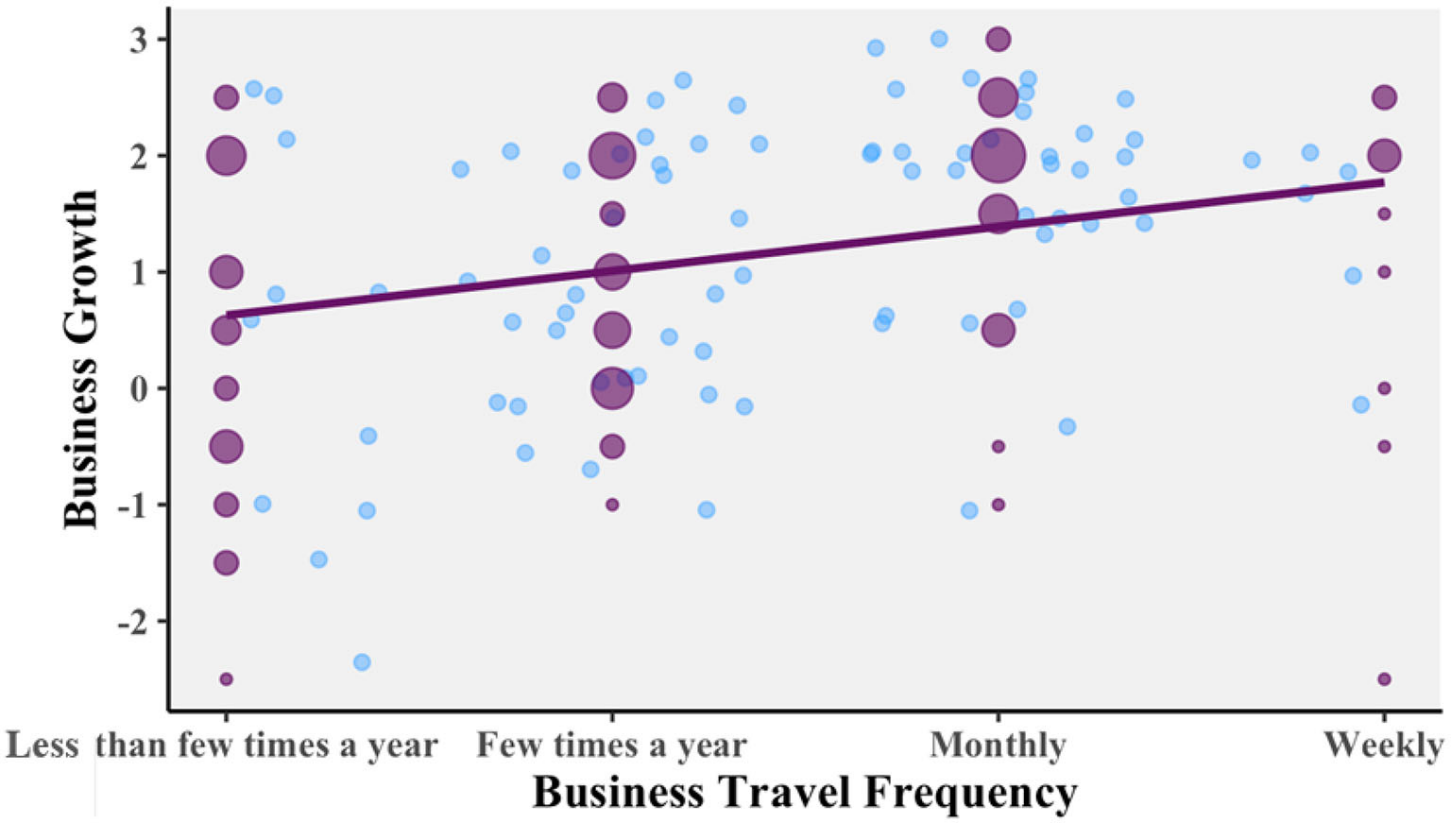
Relationship between business growth travel frequency. Scatterplot with random jitter, and Pearson correlation coefficient.

Figure 4 is a scatterplot showing one blue point for each of the respondents, based on which of the four options they chose for travel frequency and their judgment of how important travel is to their business growth. There is random jitter added to the graph, so that each point is visible. The purple dots, additionally, show the number of people at each possible response by their size. The purple line shows the linear relation between the two variables. Analysis of this supports hypothesis 1, as there is a significant and positive correlation. Pearson’s product-moment correlation *R* = 0.299, 95% CI = 0.113 – 0.465, *t*(102) = 3.164, *p* = 0.002053.

The interviewees all believed that “business travel has been essential for achieving the results” they have; and two thirds of them also endorsed the idea that business travel increases business growth. There were several discussion points which provided an explanation of their rationale. For instance, one interviewee dealt “with a network of business partners across the world. It is very important to establish that personal relationship with their senior executives… and that requires a certain amount of face-to-face activity.” (Interview 1).

Interviewers also raised complicating factors around business travel. One interviewee mentioned the problem with regular travel and maintaining office relationships and workload. The idea of forging a better balance between travel for business and other priorities was mentioned by several respondents. The stresses of business travel were notable, but no consensus emerged from the group regarding its suitability for them as employees.

Regarding virtual teams, one of the interviewees said that to develop a team located in different sites, the sense of belonging to a team and being together developing relationships and trust, is key to success. And face-to-face contact remains by the best means to establish that rapport. However, interviewees supported the idea of Oertig and Buergi (2006) that irregular physical meetings, perhaps only annually, is enough to keep a virtual team working efficiently. Therefore, while face-to-face contact is needed within team-based environments, it can be kept to a minimum without significant drawbacks.

The overall message from the interviewees, supporting most of the literature mentioned above, is that achieving a balance between travel for business, other work priorities, and personal wellbeing is the goal to optimize employee and corporate prosperity.

### Corporate culture

**Ha(4), “corporate culture is the predominant reason business travel is undertaken as frequently as it is”.**

**Figure 5.**
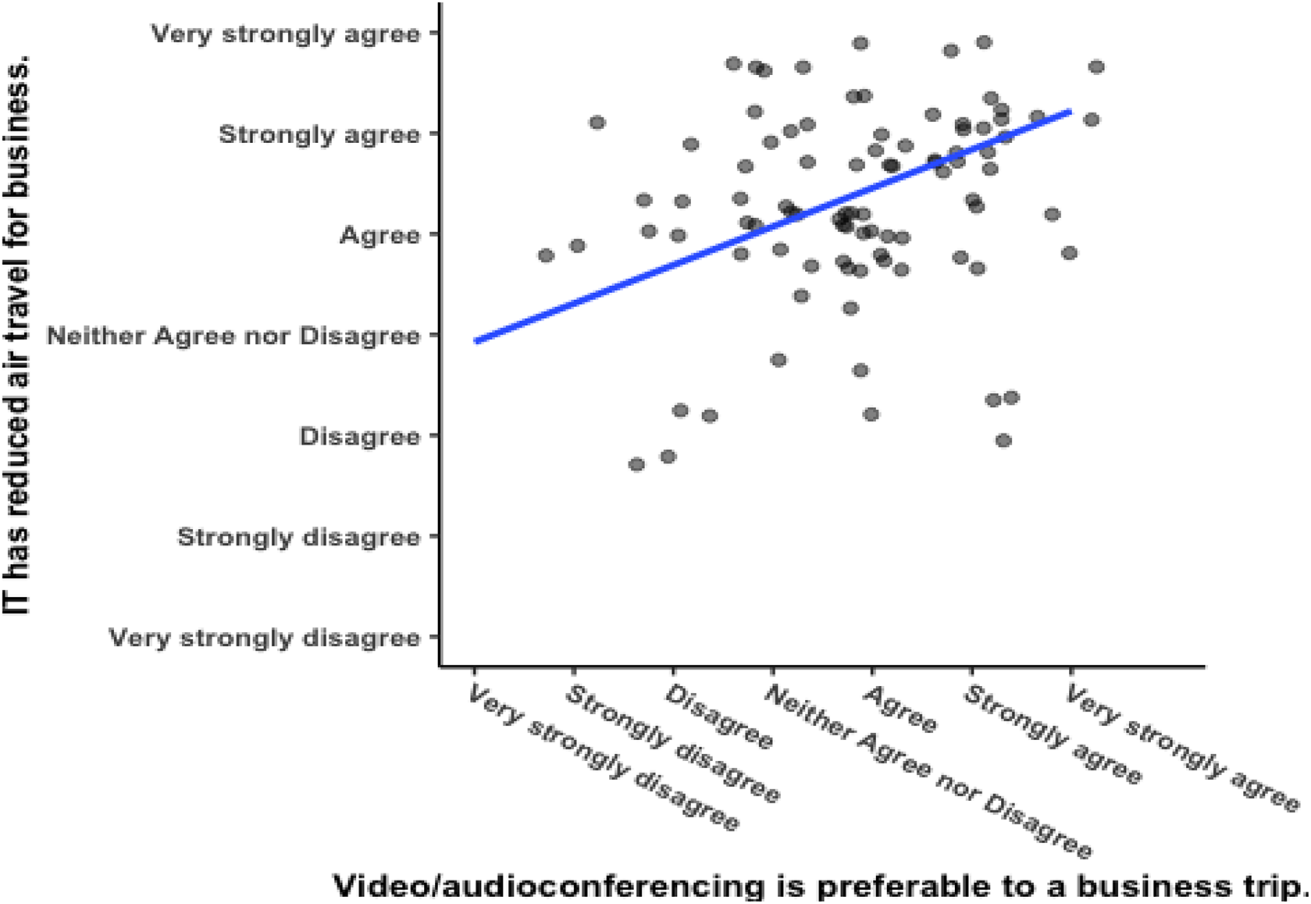
Responses to questions about remote meetings. Scatterplot and Pearson correlation coefficients.

**Figure 6.**
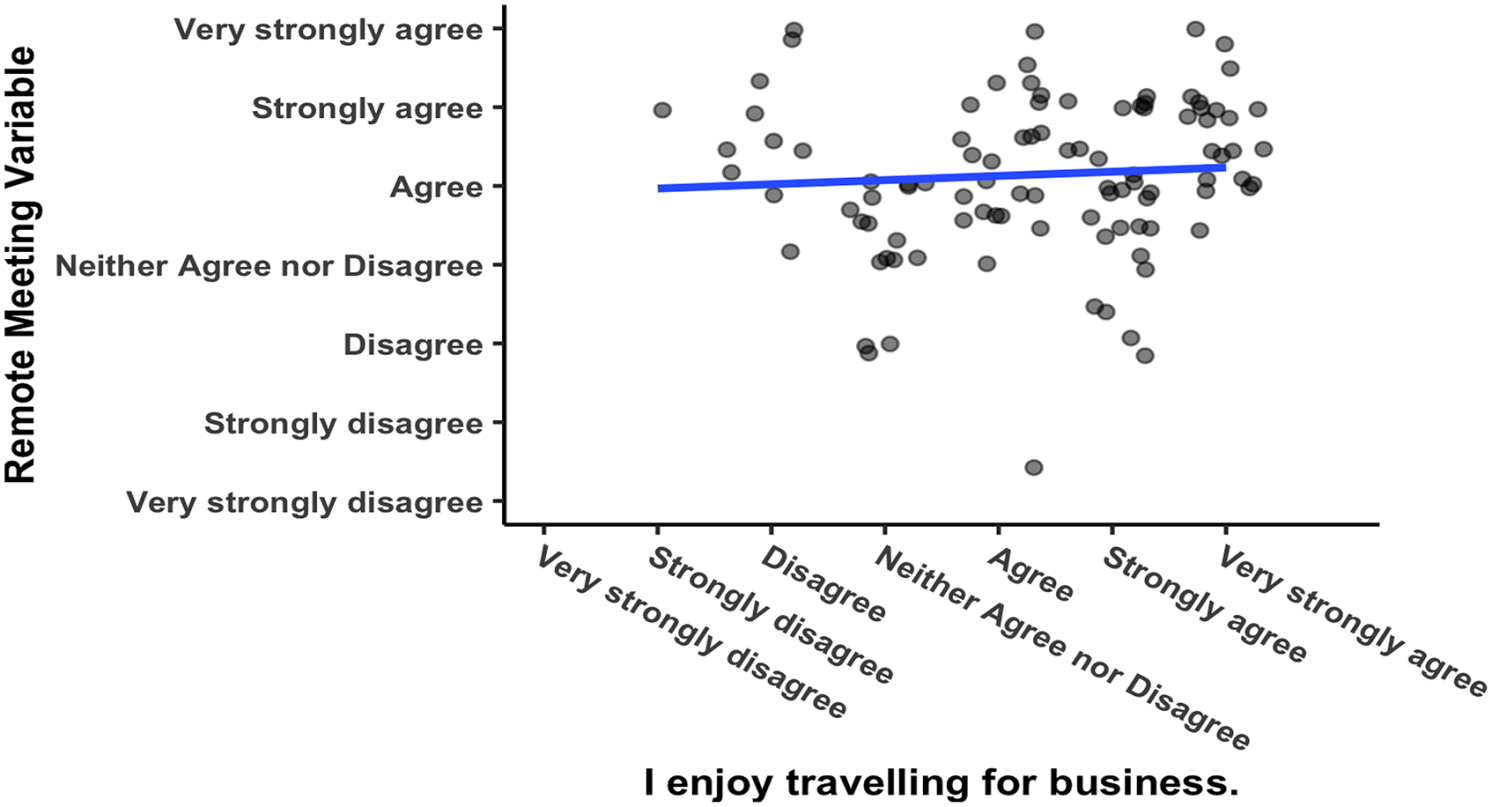
Responses to questions about meeting culture.

Figure 5 shows a significant positive correlation between responses to the two questions that were asked regarding corporate culture (see Table 1 for the questions), with Pearson’s product-moment correlation *R* = 0.51, 95% CI = 0.282 – 0.592, *t*(102) = 5.1003, *p* = 0.000002. Respondents from corporations with a meeting culture that emphasizes meeting face-to-face would be expected to report greater enjoyment of business travelling. For one, they were hired by the corporation, so they are likely to embody the culture to begin with. Secondly, as mentioned above in the method section, spending time in a culture in which business travel as a norm makes it more likely that someone will believe they enjoy business travel. The researcher compared the responses to this question against the variable “remote meeting” and found that they were not significantly correlated (see Figure 6), Pearson’s product-moment correlation *R* = 0.062, 95% CI = −0.133 – 0.253, *t*(102) = 0.626, *p* = 0.533. Enjoyment of business travel and a preference for videoconferencing are two separate constructs.

The interviews gave some further light into this disconnect. It was said that better sharing of the information on how, where, who, and what for, business travel is occurring, would mean that the travel can be consolidated as smart travel and reduce the amount of unnecessary travelling. A problem with this is that people might not be willing to share all the intentions of business travelling. An additional aspect to consider is that training to use appropriate technology is required to make people use them. If the videoconferencing equipment were advanced, secure, and well supported then this would reduce travel for business. Given COVID-19 standards are emerging and gaining broader support in this area ( Kagan, Alpert, & Fire, 2020; Weil & Murugesan, 2020).

For the corporation, interviewees found several benefits for travel. These included: understanding the local market, interaction with affiliates, motivation, better results, build relationships, business growth, generate more business, and trust building. It allows people to build up relationships, make decisions quicker, and avoid the infuriating ‘ping-pong messaging’ often seen in remote communication. Business interactions involve a myriad of nonverbal cues (including body language) and therefore face to face meetings have the advantage of capitalizing on that. Yet for the individual engaging in business travel, the free discussion was predominantly negative. It was voiced that traveling for business is exhausting and that it is not beneficial for everyone; the load on businesspeople with families was mentioned several times, as was health.

The interviewees had several inputs of value for a discussion of broader corporate culture:

• Need for CSR policies embedding environment and business travel.
• Sustainability to be part the corporate values.
• Executives to be role models to make the message stronger.
• Internal communication improvement & clearer strategy/guidance.
• Practice and recognition to become part of the company culture.
• Allocation of a measuring manager (KPI).
• CO _2_ emissions monitoring and measuring.
• Willingness to pay for CO _2_ emissions (green taxes).
• In the job description, the travel percentage needs to be defined and indicate how to be measured.
• To be successful developing a remote team it is key the sense of belonging and being together developing trust and relationship.
• Increasing ICT (information and communications technology) tools and training for optimal usage.
• Paying for CO _2_ emissions.
• The use of electrical cars as a company cars policy.
• Smart traveling awareness and implementation 탖 consolidation.
• Definition of electrical cars as a company car.
• Legal regulations from the local authorities.
• Clearer strategy and corporate guidance.
• General reduction of business travel.
• To be always traveling would be a disadvantage to the organization and subsequently, balance is the goal for the employee and corporate prosperity.

### Corporate social responsibility

**Ha(5), “as corporate social responsibilities policies linked to environmental awareness (from the corporate) increases, business travel decreases”.**

The survey asked the following question directly:

• Q19) If corporations pursued pro-environment corporate social responsibility policies, would business travel increase or decrease?

Of 102 responses to that question, 93 (91%) responded that business travel would decrease in such a situation, and 9 (9%) responded that it would increase. Therefore, 91% of the respondents endorsed Ha(5) in the hypothetical. A similar result was achieved in the interviews – 14 of the 15 respondents (93%) endorsed the question. While this is a hypothetical framing of the question, which comes with limitations discussed above, it highlights something that future research might want to pick up on, for instance by studying the link between awareness, CSR policies and business travel in practice.

One of the interviewees indicated that if pro-environment policies were implemented, travel for business would be reduced in the short term only; the corporation would need to keep investing in a pro-environment program. To succeed, it would need to be implemented and embedded as part of the culture of the corporation. Another respondent said that if a company was environmentally friendly with the usage of paper, water waste, recycling, etc., then it must also consider the impact of business travel on global warming. Physically travelling for business purposes needs to be seen not as purely a sunk cost in travel expenditure. It must be measured in terms of environmental impact as part of the values of the corporation. Without a quantified measure such as that, pro-environment policies would tend to be unsustainable in the long run. These kinds of policies only work when supported and believed in by senior leadership.

The interview brought out an important point regarding CSR policy. Only four of the 15 interviewees knew that they did already have a policy in place. Of these, only two of the four said the policy referred to travel for business, one said the environment, and the other to neither of them. One of them indicated that the company at the focus of the company’s own case study is moving from Scope 2 up to Scope 3. Scope 2 are indirect emissions from the generation of purchased energy (Greenhouse Gas Protocol Corporate Standard (GHGPCS) Value Chain Emission) whereas Scope 3 emissions include all indirect emissions that occur in the value chain of the reporting company, including both upstream and downstream emissions. All four belonged to the more senior group of employees interviewed.

Four of the other interviewees (27%) replied that the company did not have a CSR policy. Seven did not know. Therefore, it can be assumed that related internal information is not distributed in a clear and homogeneous manner within the business for all employees. It would seem evident that internal communications are an area for the business to focus upon in the future. Other literature indicates that this finding is far from unusual ( Yue, Men, & Ferguson, 2020), but given comments above about the importance of communication from senior leadership to embed pro-environment policies, it is of note.

It is also worth noting that travel policies were in place within the company to control expenditure, hotel grade selection, car hire price range, weekend allowances, flight price range (related to business class, economy, or premium economy) depending on the length of the flight, etc., but not related to any environmental concerns or CSR. Again, this is far from unusual.

### Additional conclusion – relationship between environmental knowledge, and willingness to pay green tax

There were three items on the questionnaire regarding environmental knowledge (see Table 1).

Responses to these three items were correlated with willingness to pay both corporate and individual green tax (see Tables 5 and 6).

**Table 5.** *R* values for correlations between self-reported environmental awareness and willingness to pay corporate green tax.

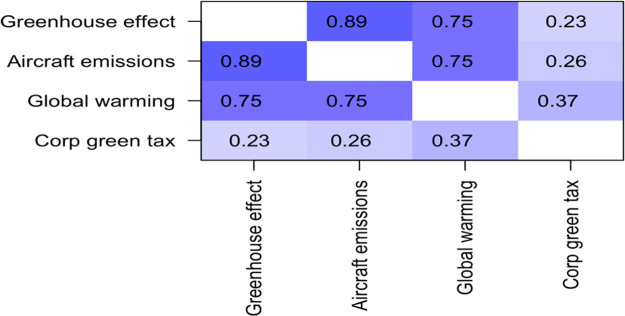

Unsurprisingly, the three questions about environmental awareness were highly positively correlated with one another, with R values ranging from 0.75 to 0.89. The correlation with willingness to pay corporate green tax were also positive, and statistically significant, with *R* values of 0.23, 0.26, and 0.37 (see Table 6).

**Table 6.** *R* values for correlations between self-reported “environmental awareness” and “willingness to pay individual green tax”.

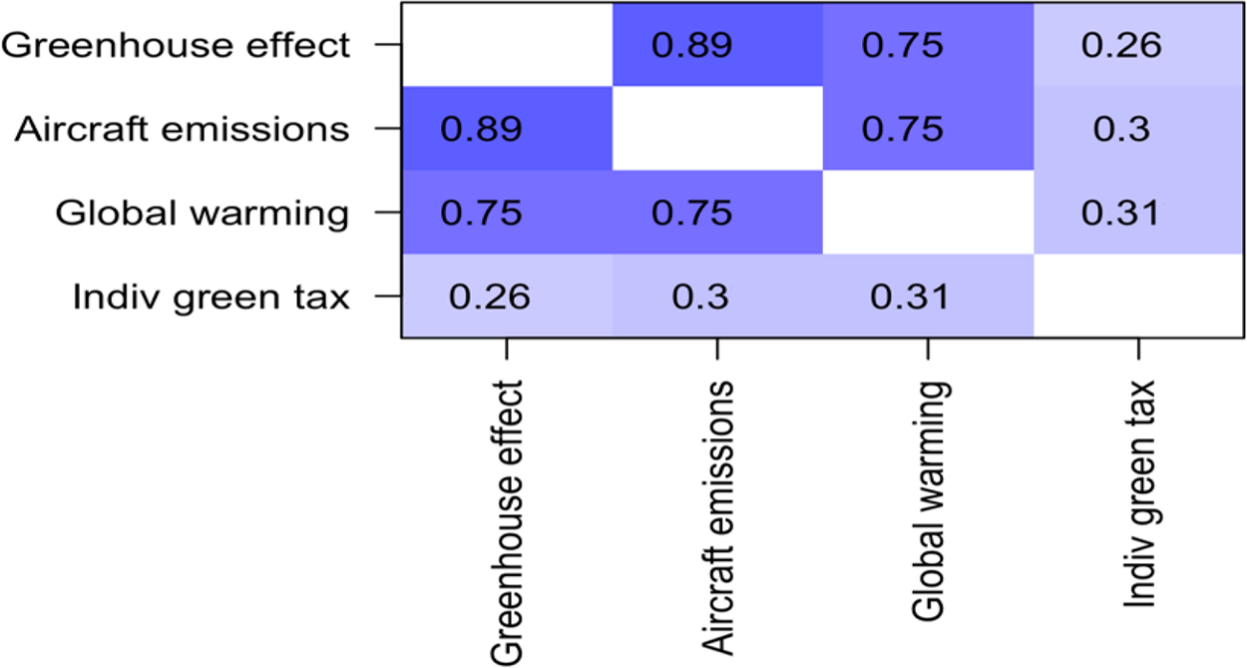

The correlations with “willingness to pay individual green tax” were also positive, and statistically significant, with *R* values of 0.26, 0.30, and 0.31. In this study, environmental knowledge was correlated with willingness to act, both as an individual and corporation.

## Discussion

While business travel is a global issue, many of the cultural aspects discussed above have a local element. The study was focused upon the Zurich airport area in Switzerland, a country which is renowned for its environmental awareness. It may be that in other geographical regions, different results would be found which would add to the body of knowledge regarding the relationship between the major constructs of the study.

Perhaps more significantly, the pandemic of COVID-19 has placed a chronological limitation on this study, and indeed any recently conducted study regarding business travel (along with many other aspects of life). While the environmental imperative has not changed, many other influences on business travel and the corporate culture around it have altered markedly since the research was carried out. Therefore, this study will provide a crucial point of comparison for later work looking at the same topic: a clear ‘before’ point to assess the impact of COVID-19 on business travel habits and opinions.

Within those limitations, and others mentioned above in the methods section, this article adds to the evidence base that a more targeted and specific approach to business travel would enhance employee wellbeing and reflect our knowledge of the environmental impacts of carbon emissions ( Poom, Orru, & Ahas, 2017). The survey demonstrated that neither individual nor corporate awareness leads, on its own, to reduced business travel. What is needed, as other literature has argued, is both a cultural shift and the integration of CSR into business travel policies ( Koonin, 2020; Hepburn et al., 2020; Kuzemko et al., 2020). In the survey reported above, 91% of those surveyed believed that a greater integration of CSR policies into business travel would lead to a decline in business travel.

In corporations which have a pro-business travel culture, one might expect more travel will be taken and less videoconferencing or remote meetings will be arranged. This is justified by those corporations based on business growth, which as this research has shown using both quantitative and qualitative methods, is considered to be facilitated by face-to-face meetings, particularly in the early stages of many business relationships. It has been argued before that physical co-presence is important for building trust quickly ( Castells, 2010; Faulconbridge, Beaverstock, Derudder, & Witlox, 2009; Lo *et al*., 2013), as was reported by the interviewees with reference to virtual teams above. This article, particularly through the interviews, provides further evidence for that.

However, for corporate leaders, the interviews elicited several valuable ideas for reconsidering corporate culture in a more wholistic and nuanced way. Recognizing the value of business travel and face-to-face discussion does not necessarily mean supporting business travel in every context. If sustainability is embedded as a key part of the corporate value set and acted on by executives to make that message stronger, business travel would reduce. The interviewees raised the importance of clear communication, including prior to hiring around travel expectations, and within the company around an integrated sustainability and travel strategy. As well as the necessity of resources for ICT training.

The overriding sentiment was that these factors must be embedded into business culture. COVID-19 has undoubtedly provided the environment a break from air and noise pollution, which is notable from datasets in the public domain. The pandemic has also done plenty to illustrate just how much people can change their behaviors, when they are highly motivated or forced to do so. Taking up some of the issues listed above would enable corporations to facilitate both a business culture that contributes positively to social and environmental issues, and which takes care of its employees. Traveling for business is hard on the body mentally and physically, as well as time consuming ( Bunn & Johnson, 2019; Mäkelä & Kinnunen, 2018). Therefore, it is sensible to conclude that videoconferencing should be used more where the downsides of travel are not offset by significant growth probabilities. There is significant relationship between business growth and business travel as face-to-face meetings are essential to build trust and relationships. However, travel can be significantly reduced, interviewee suggested by more than 50% as indicated the interviewee 2, and depending on the purpose of the meeting, it may not be necessary, for instance for standard topics ( Bripi, 2019). In matrix organizations once a year face to face meeting is sufficient to run virtual teams ( Oertig and Buergi, 2006).

The case study found the willingness to pay both corporate and individual green taxes was positive and statistically significant. Further replication of the study would be desired to conclusively determine if these significant values could be generalized beyond the study population. Particularly so as Switzerland is renowned for its environmental awareness, which may make the population atypical on a European, and indeed global, level. Nevertheless, the results of the study add to a body of evidence that there is willingness to pay green taxation if the Swiss government were to implement it ( Bernhard, 2020; Maxim, Zander, & Patuelli, 2019).

Additionally, it is evident that corporate communication on environmental issues is currently suboptimal. The case study demonstrated that even in an organization which is making positive moves towards Scope-3 emissions considerations, the communication of that desire within the company, was lacking. A notable absence from the interviews conducted during the case study, were the United Nations 2030 Sustainable Development Goals, which went entirely un-mentioned by participants. Government policies must develop mechanisms to highlight this when implementing CSR regulations ( Rela *et al.,* 2020; Pinzone *et al.,* 2019).

With COVID-19 providing a push towards the utilization of videoconferencing systems, many individuals and corporations are being forced to develop the skillsets needed to make best use of technology. Companies are investing in appropriately secure videoconferencing systems, server, and broadband technologies. Will restrictions on travel be a long-term part of life? Will businesses make the conscious choice to embrace virtual communication where it is most appropriate? Or will business travel return to something like the pre-COVID normal, given time? Fundamental questions like this remain unresolved. Further research will thus be essential to assess the longer-term implications of COVID-19 in this area.

## Data availability

### Underlying data

UK Data Service: Environmental Perception of Global Business Travel by Swiss Companies in the Zurich Airport Area, 2019-2020. https://doi.org/10.5255/UKDA-SN-854930 ( Echeverria-Arrondo & Wolfs, 2021).

This project contains the following underlying data:

• Survey individual raw data.xlsx
• Survey master data from Qualtrics.csv
• Case study interview results overview.xlsx
• Case study interview transcripts.pdf

### Extended data

UK Data Service: Environmental Perception of Global Business Travel by Swiss Companies in the Zurich Airport Area, 2019-2020. https://doi.org/10.5255/UKDA-SN-854930 ( Echeverria-Arrondo & Wolfs, 2021).

This project contains the following extended data:

• Case study interview consent form.pdf
• Survey questionnaire in English and German.pdf
• Survey report from Qualtrics 2019.pdf
• Case study interview schedule August - September 2019.pdf
• Case study introduction letter.pdf
• Non-disclosure Agreement for the case study.pdf
• Qualitative questionnaire - case study.pdf
• Chapter 4 from the dissertation - quantitative - research findings and discussion.pdf
• Chapter 5 from the dissertation - qualitative - case study Swiss pharma company.pdf

Data are available under the terms of the Creative Commons Attribution 4.0 International license (CC-BY 4.0).
