# Supplementary material for: Environmental perceptions of global business travel by Swiss companies in the Zurich airport region
Source: F1000Res. 2022 Dec 23;10:925. Originally published 2021 Sep 15. [Version 3] doi: 10.12688/f1000research.54862.3 (PMC9860200; doi:10.12688/f1000research.54862.3)
Supplement: Peer review history

# Reviewer report: Reviewer response for version 3

**DOI:** 10.5256/f1000research.141624.r158547

**Published:** 2023-01-20

**Authors:** Stefan Baumeister

**Affiliations:** 1 University of Jyväskylä, Jyväskylä, Finland

I have read the latest version of the below mentioned article and I am satisfied with the all the changes the authors have made to their manuscript. As such I would recommend for indexing of the manuscript in its current form.

Is the work clearly and accurately presented and does it cite the current literature?

Partly

If applicable, is the statistical analysis and its interpretation appropriate?

No

Are all the source data underlying the results available to ensure full reproducibility?

Partly

Is the study design appropriate and is the work technically sound?

Partly

Are the conclusions drawn adequately supported by the results?

No

Are sufficient details of methods and analysis provided to allow replication by others?

Yes

Reviewer Expertise:

NA

I confirm that I have read this submission and believe that I have an appropriate level of expertise to confirm that it is of an acceptable scientific standard.

# Reviewer report: Reviewer response for version 3

**DOI:** 10.5256/f1000research.141624.r158548

**Published:** 2023-01-16

**Authors:** Mònica Guillen Royo

**Affiliations:** 1 CICERO Center for International Climate Research, Oslo, Norway

I thank the authors for their careful revision of the paper and I declare that at this stage, I do not have further comments to make.

Is the work clearly and accurately presented and does it cite the current literature?

No

If applicable, is the statistical analysis and its interpretation appropriate?

No

Are all the source data underlying the results available to ensure full reproducibility?

Yes

Is the study design appropriate and is the work technically sound?

Partly

Are the conclusions drawn adequately supported by the results?

No

Are sufficient details of methods and analysis provided to allow replication by others?

Yes

Reviewer Expertise:

Sustainable consumption, mixed methods research, wellbeing studies, economics of happiness, human scale development

I confirm that I have read this submission and believe that I have an appropriate level of expertise to confirm that it is of an acceptable scientific standard.

# Reviewer report: Reviewer response for version 2

**DOI:** 10.5256/f1000research.133483.r136743

**Published:** 2022-10-17

**Authors:** Mònica Guillen Royo

**Affiliations:** 1 CICERO Center for International Climate Research, Oslo, Norway

I thank the authors for the thorough revision of the paper, which has improved in structure and clarity. 

 Some changes can still be made to strengthen clarity and robustness. 

- The wording of the objectives, 'we have to', 'we need to' points to a distinction between structural factors and intrinsic factors. It is unclear whether the paper is focused on this distinction and thus the objectives could be reframed to better reflect the title and the hypotheses of the study.
- The hypotheses are not clearly linked to the references included in the literature background, either make explicit from which of the references they derive or mention right before or after each hypothesis the studies that suggest the presence of a significant relationship or the lack of it.
- When describing the questionnaire there is a sentence that might need rephrasing: ' There are several hypothetical questions within the set, in which the individual gives their opinion regarding something regarding the business they work at. '
- The list of interviewees' inputs when discussing Ha(4) should be summarized in one paragraph that offers an interpretation of these inputs to the reader. For example, they could be presented as related to the company, the business sector or the governance level (local and national regulations or taxes for instance).

Overall, this is a very much improved version of the article that deems indexing provided some small changes are implemented.

Is the work clearly and accurately presented and does it cite the current literature?

No

If applicable, is the statistical analysis and its interpretation appropriate?

No

Are all the source data underlying the results available to ensure full reproducibility?

Yes

Is the study design appropriate and is the work technically sound?

Partly

Are the conclusions drawn adequately supported by the results?

No

Are sufficient details of methods and analysis provided to allow replication by others?

Yes

Reviewer Expertise:

Sustainable consumption, mixed methods research, wellbeing studies, economics of happiness, human scale development

I confirm that I have read this submission and believe that I have an appropriate level of expertise to confirm that it is of an acceptable scientific standard, however I have significant reservations, as outlined above.

# Response

**Published:** 2022-11-27

**Authors:** Ignacio Echeverria Arrondo

**Affiliations:** SBS Swiss Business School, Switzerland

Dear reviewer,

 Thank you again for your detailed feedback.

 In the new version, the ethics clearance section has been removed and the wording of the objectives has been amended, as suggested. An additional section was added to explain the literature base behind the development of each hypothesis tested in the study.  A sentence regarding the hypothetical nature of some of the questions within the survey was re-worded for clarity. And the list of interviewees’ inputs was summarised across three short paragraphs to address comments related to policymakers, business leaders and corporate culture.

 In the hope that these edits will work to your satisfaction, I would greatly appreciate if you could review the article and its rating, once the new version has been published in the next couple of days.

 Kind regards

 Ignacio Echeverria Arrondo

# Reviewer report: Reviewer response for version 2

**DOI:** 10.5256/f1000research.133483.r136744

**Published:** 2022-07-22

**Authors:** Stefan Baumeister

**Affiliations:** 1 University of Jyväskylä, Jyväskylä, Finland

I would like to thank the authors for all the changes they have made to the manuscript which I feel have significantly improved the quality of the article. Nevertheless, I still feel a certain disconnect between the hypotheses presented in the methods section and the newly added background literature. It is usually common that hypotheses presented in a study are clearly connected with previous literature but in this case it is quite difficult to understand the rational behind the hypotheses based on the presented literature. I would like to encourage the authors to better link their hypotheses with their background literature by providing a separate literature section for each hypothesis.

Is the work clearly and accurately presented and does it cite the current literature?

Partly

If applicable, is the statistical analysis and its interpretation appropriate?

No

Are all the source data underlying the results available to ensure full reproducibility?

Partly

Is the study design appropriate and is the work technically sound?

Partly

Are the conclusions drawn adequately supported by the results?

No

Are sufficient details of methods and analysis provided to allow replication by others?

Yes

Reviewer Expertise:

NA

I confirm that I have read this submission and believe that I have an appropriate level of expertise to confirm that it is of an acceptable scientific standard, however I have significant reservations, as outlined above.

# Response

**Published:** 2022-11-27

**Authors:** Ignacio Echeverria Arrondo

**Affiliations:** SBS Swiss Business School, Switzerland

Dear reviewer,

 Thank you again for your detailed feedback.

 In the new version, the ethics clearance has been removed as was previously suggested. An additional section was added to explain the literature base behind the development of each hypothesis tested in the study, for clarity.

 In the hope that these edits will work to your satisfaction, I would greatly appreciate if you and/or your co-reviewers could review the article and its rating, once the new version has been published in the next couple of days.

 Kind regards

 Ignacio Echeverria Arrondo

# Reviewer report: Reviewer response for version 1

**DOI:** 10.5256/f1000research.58384.r119974

**Published:** 2022-02-16

**Authors:** Mònica Guillen Royo

**Affiliations:** 1 CICERO Center for International Climate Research, Oslo, Norway

This study uses mixed methods to study the relationship between job-related flying practices in companies around the Zurich geographical area and employees perceptions and attitudes towards flying. The paper is well-structured, and the topic is interesting and timely. Nevertheless, lack of clarity concerning the theoretical and methodological underpinnings of the study deem it unsuitable for indexing in its present form.

**General considerations**

 In general, the goal of the study is not clearly presented, and the hypothesis not explicitly grounded on a thorough literature review on the topic. The mixed-methods approach is very suitable for this type of study, but its advantages have not been explicitly discussed and it is unclear for the reader what it is that the qualitative study has added to the results of the descriptive quantitative analysis. The quantitative analysis focusses on the description of variables and a correlational study, but since the general research question is not described, the extent to which the analysis contributes to a greater understanding of corporate travel is unclear.

**Detailed comments**

 The abstract should include the main goal of the study (in the background subsection) and results should not refer directly to the hypotheses as the reader does not know what these are at the point of reading the abstract. References to the COVID-19 pandemic might be done in the conclusion but not in the background section as this study was not carried out against the backdrop of the pandemic.

 The introduction would benefit from including an explicit reference to the goal of the study. It would also be advisable to link the questions that the researcher presents as inspiring the study to a problem in society (health or wellbeing related impacts of flying), the environment (ecological impact of flying) or a gap in the literature that the study wants to fill. Additionally, it is common in academic articles to use the last paragraph of the introduction to describe the sections of the paper and their contents.

 Concerning the background literature section, this should be sufficient to justify the hypotheses presented in the Methods section. At present, it lacks enough depth. The literature on business travel is very extensive. Employees’ values and attitudes have been investigated both drawing on quantitative and qualitative methods and should feature in this section. This is also the case concerning policies to reduce air travel and their lack of effectiveness due to the many barriers and taboos concerning mobility and flying in contemporary societies. The *Journal of Air Transport Management*, the *Journal of Transport Geography* and *Transportation Research Part D* publish many studies that the authors might want to include in this section. Particularly relevant are the works by Stefan Gösling, Scott Cohen and James Higham. Additionally, Sahakian et al. (2021) ^1^ have recently published an article on air travel in (Genève) Switzerland based on document analysis and the analysis of workshop data.

 In the methods section, some details about ethics clearance and piloting might not be necessary, whilst information about the strategy followed to analyze qualitative data is missing. It is also unclear the bias that the authors would create if they had interviewed respondents themselves. Additionally, it is important to clarify how multistage sampling was applied, if ‘clusters were formed of companies with which the researcher had a pre-existing relationship’, what type of companies were left out? What was the structure of the clusters and how could this sampling strategy be presented as having a random component in it? Moreover, how were the answers to the ‘global business travel’ questions coded, or alternatively were they open questions? Table 1, would be better presented as a descriptive statistics table, where variables are presented in terms of the question, the response scale and measures of central tendency and dispersion.

 When presenting the results, it would be useful to uniformize the way the data is presented, using the same type of figures or tables across subsections. In addition, when scales are formed (by adding up the scores of more than two variables) it would be advisable to present the value of Cronbach’s alpha, which would indicate whether there is strong or weak internal consistency of the construct. Furthermore, there seems to be a potential source of confusion concerning the section on business travel and environmental awareness, as it is difficult for the reader to discern why a measure for ‘social responsibility’ is created but later, findings are presented in terms of individual vs. corporate awareness.

 Another concern in this section is related to one of my previous general comments, why is the qualitative information important? How does it help in understanding the relationship addressed in this section? The biggest challenge here is that the goal of the paper is unknown, so the reader cannot easily follow what the article is trying to achieve. If the goal was to understand how environmental perceptions affect business travel, then one would expect regression analysis to be used to explain the determinants of business travel frequency for example, but this is not done here probably because of the small sample size and the lack of availability of socio-demographic variables.

 Finally, Ha (5) is difficult to investigate drawing on a variable that captures results from a hypothetical question. Studying this hypothesis would require having data about the extent to which corporate social responsibility policies are in place in each company, which does not seem to be the case in the survey of this study.

 One expects the discussion section to go back to the literature described in the second section to discuss the results of the study. Listing the topics raised by interviewees does not appear to add much to the discussion and might be removed. The paragraph on videoconferencing refers strongly to the COVID-19 pandemic, and although this might be a topic for further research cannot be used here to draw conclusions on policy as the study was carried out before the COVID-19 outbreak. An important finding of the study is the one concerning corporate communication. Probably the article and the analysis would increase their relevance if they had been articulated around this very important topic.

Is the work clearly and accurately presented and does it cite the current literature?

No

If applicable, is the statistical analysis and its interpretation appropriate?

No

Are all the source data underlying the results available to ensure full reproducibility?

Yes

Is the study design appropriate and is the work technically sound?

Partly

Are the conclusions drawn adequately supported by the results?

No

Are sufficient details of methods and analysis provided to allow replication by others?

Yes

Reviewer Expertise:

Sustainable consumption, mixed methods research, wellbeing studies, economics of happiness, human scale development

I confirm that I have read this submission and believe that I have an appropriate level of expertise to state that I do not consider it to be of an acceptable scientific standard, for reasons outlined above.

## References

[1] : Flying Less for Work and Leisure? Co-Designing a City-Wide Change Initiative in Geneva. *Urban Planning* .2021;6(2) : 10.17645/up.v6i2.3911 299-313 10.17645/up.v6i2.3911

# Response

**Published:** 2022-04-24

**Authors:** Ignacio Echeverria Arrondo

**Affiliations:** SBS Swiss Business School, Switzerland

Dear reviewer,

 Thank you for your detailed feedback, and please excuse the delay in responding.

- Abstract – in the new version, the background section was rephrased to include the goal, and the results are not referring directly to the hypotheses, as suggested.
- Introduction – an explicit reference to the goal of the study was added as well as an outline of the article’s structure, as suggested. Additions were made to position the article more clearly as contributing to the evidence base around CSR, international business travel, and employee wellbeing.
- Background literature –  references were added, after revisiting notes from the doctoral thesis from which this article emerged and reviewing more recent work on employee responses to air travel, as well as per your suggestions. Several edits were made to ensure it is clear to the reader that the article provides evidence to demonstrate, largely in line with previous literature, that awareness is insufficient; action must be taken. Some suggested actions which emerged from the interviews and research were added in a new paragraph in the conclusion.
- Methodology – in the opinion of the main author, piloting, NDA and ethical clearance are important in this case. Perhaps the name of the interviewer and alternative software could be removed.
- Results – this section also contains several edits. Clearer figure captions. A reduction in the repetition of the survey questions. An explanation of how the combined variables were created. A few quotes from the interviews were added, and the broader “inputs of value for a discussion of broader corporate culture” from the interviews, which was in the conclusion in the previous version, have been brought up in the results section. Concerning the H(5) justification for the usage of hypothetical questions within the set, it was explained in a new paragraph as part of the discussion of the quantitative methodology, with reference to appropriate literature. References and evidence have been added.
- All other comments have been partially implemented where possible.

In the hope that these edits will work to your satisfaction, I would greatly appreciate if you could review the article and its rating, once the new version has been published in the next couple of days. 

 Kind regards

 Ignacio Echeverria Arrondo

# Reviewer report: Reviewer response for version 1

**DOI:** 10.5256/f1000research.58384.r94451

**Published:** 2021-09-28

**Authors:** Stefan Baumeister, Sami El Geneidy, Maija Lähteenkorva

**Affiliations:** 1 University of Jyväskylä, Jyväskylä, Finland

This research paper addresses an interesting and timely topic as it studies the environmental perceptions of global business travelers in Switzerland. While I very much enjoyed reading the paper and found the findings it provides interesting and to some extent also novel, I must also say that I identified some major short-comings that would need to be addressed before I could recommend indexing this paper.

 A major problem I see is with the sample size which is only 104 survey participants. As the authors pointed out correctly, for this population size the minimum sample size should be 384 participants. As it is common that not all invited potential participants will respond to a survey, I am wondering why you only sent out invitations to 400 individuals when you acknowledge a required minimum of 384 participants? Nevertheless, given the fact that you conduct a mixed-methods approach with in-depth interviews, the low amount of survey participants could be accepted. However, you would need to clarify in your paper that such a small amount of participants significantly reduces the validity of your study and the conclusions drawn from the sample. In addition to that, not collecting demographic data is another major short-coming of this study. This cannot be made up by assuming the demographics. Such information should therefore not be presented in the paper as it could misguide the reader.

 Another major issue with this study is the lack of literature to back up the hypotheses presented. Although the authors mentioned in the introduction that hypotheses were developed based on secondary literature, these sources were not presented sufficiently in the paper. I would therefore encourage the authors to add a well-developed literature section to the paper that justifies the presented hypotheses clearly, backing them with existing literature. Just listing the hypotheses at the beginning of the methods section isn’t sufficient enough.

 In the results section I don’t see any need to present/repeat the survey questions in such great detail but to rather focus on the actual results. Also focus should only be placed on the most relevant findings. Please revise the results section accordingly.

 Finally, I find that the discussion section is focusing mainly on the findings of the case study but more or less neglects the rich findings from the survey. As this is a mix-methods approach, I would like to see more discussion on the findings of both studies instead of only the ones from the interviews.

 Last but not least I see in these findings also great potential for policy recommendations the authors could make based on the study. Therefore, I would encourage the authors to add a section on policy recommendations to the discussion section at the end of the section.

 In addition to these major concerns I have some further detailed comments for the author to considered during the revision:

 In the results section of the abstract, I would recommend to leave out the exact results from the hypotheses testing as this is not common practice and also difficult for the reader to assess unless he/she is familiar with the hypotheses. The abstract should be a alone-standing document that a reader can fully assess without the need to read the paper as such. Instead of the precise results of the hypotheses testing I would recommend the authors to focus on the actual results the hypotheses tests provided.

 In the introduction, please rephrase the first sentence as it is confusing. What do you mean with “coronavirus disease 2019 pandemic”? Further in the first sentence of the third paragraph you cite two sources but one as an in-text citation and the other at the end of the sentence. Is there a particular reason for that as this is not common practice?

 It is very nice to see that the idea for this study was based on a critical assessment of travel behavior by the author himself. However, perhaps you could still use a more general and passive voice when presenting those arguments as the study otherwise appears more like a narrative which it is apparently not.

 In the background literature section, you cite in the first sentence the Harvard Business Review (2009). Instead of the journal name could you please provide the name of the author of this publication? In the same paragraph you also use the author Tani’s first name initial which should be avoided in in-text citations.

 In the objectives section I would leave out the word “simple” when presenting the research questions as these are by far not simple questions you are attempting to answer with this study.

 Some aspects in the methods section are in my opinion described in too great detail and not necessarily relevant for the reader to understand how the study was conducted or for researchers to replicate the study, among those were:

- The questionnaire testing process is described in too much detail
- Alternative software that could have been used to conduct the survey
- The name of the person conducting the interviews and the training he received for that
- Details on the use of NDA and its storage
- Information on ethical approval

Instead, the data analysis could have been described in slightly greater detail.

 The first two paragraphs of the discussion section are more or less repeating the background and purpose of the study instead of really discussing the actual results. Perhaps those paragraphs could be shortened or left out entirely?

 The results of the interviews of value for a discussion of broader corporate culture presented in the discussion section are really interesting. Nevertheless, these are results and should not be presented in the discussion section but rather in the results section. Also, instead of presenting these findings in bullet points you might consider presenting them in a table or graph which might be more appealing to the reader.

 Further comments and an annotated version of the manuscript from my co-reviewers can be found here: Sami El Geneidy, Maija Lähteenkorva.

Is the work clearly and accurately presented and does it cite the current literature?

Partly

If applicable, is the statistical analysis and its interpretation appropriate?

No

Are all the source data underlying the results available to ensure full reproducibility?

Partly

Is the study design appropriate and is the work technically sound?

Partly

Are the conclusions drawn adequately supported by the results?

No

Are sufficient details of methods and analysis provided to allow replication by others?

Yes

Reviewer Expertise:

Climate Change Mitigation, Sustainable Consumption, Corporate Responsibility, Transportation Sector, Environmental Labels, Aviation Industry, Electric Aviation, Carbon Footprint Analysis, Modal Shift, De-Growth, Sustainable Tourism, Climate Change Adaptation

We confirm that we have read this submission and believe that we have an appropriate level of expertise to state that we do not consider it to be of an acceptable scientific standard, for reasons outlined above.

# Response

**Published:** 2022-04-24

**Authors:** Ignacio Echeverria Arrondo

**Affiliations:** SBS Swiss Business School, Switzerland

Dear reviewer,

 Thank you for your detailed feedback and please excuse the delay responding, as I was waiting for more reviews.

- Methodology – as for the sample size, the minimum of 400 respondents (above 384) was requested for the quantitative section of the dissertation from which this article has been extracted, to be accepted (Mugenda and Mugenda 2012). The number of valid survey participants was 104. A comment has been added in the results section; ‘while this sample was sufficient for this mixed-methods study, it is below the 384 required for the quantitative data to be taken as statistically representative of the population being studied. Future researchers may want to replicate this study to a larger sample, and also to take account of the advice in van Mol (2015) and provide repeated digital reminders for potential participants. To prevent lengthening the quantitative survey, which might have further impacted the response rate, demographic data was not collected’.
- Methodology –  the demographic estimated data has been removed as suggested.
- Background literature – more sources have been added to back up the hypotheses.
- Results – survey questions have been removed as suggested.
- Abstract – the results section has been amended although the precise results are still included. In the conclusion ‘research for this article was undertaken prior to COVID-19, so future research will be necessary to investigate the new context’ has been added.
- Introduction – ‘corona disease 2019 pandemic’ has been rephrased. Also, in-text citation has been amended.
- Literature background – Tani’s first name initial has been removed, as suggested.
- In the objective section, the word ‘simple’ has been removed, as suggested.
- In the opinion of the main author, the questionnaire tests, the NDA and ethical approval are important. Perhaps the name of the interviewer and alternative software could be removed as suggested.
- The results of the interviews have been moved from the discussion to the results section.
- All other comments have been partially implemented where and if possible. 

In the hope that these edits will work to your satisfaction, I would greatly appreciate if you and/or your co-reviewers could review the article and its rating, once the new version has been published in the next couple of days. 

 Kind regards

 Ignacio Echeverria Arrondo
