# Supplementary material for: Environmental perceptions of global business travel by Swiss companies in the Zurich airport region
Source: F1000Res. 2021 Sep 15;10:925. [Version 1] doi: 10.12688/f1000research.54862.1 (PMC9860200; doi:10.12688/f1000research.54862.1)
Supplement: Peer review history

# Response

**Published:** 2022-04-24

**Authors:** Ignacio Echeverria Arrondo

**Affiliations:** SBS Swiss Business School , Switzerland

Dear reviewer,

 Thank you for your detailed feedback, and please excuse the delay in responding.

- Abstract – in the new version, the background section was rephrased to include the goal, and the results are not referring directly to the hypotheses, as suggested.
- Introduction – an explicit reference to the goal of the study was added as well as an outline of the article’s structure, as suggested. Additions were made to position the article more clearly as contributing to the evidence base around CSR, international business travel, and employee wellbeing.
- Background literature –  references were added, after revisiting notes from the doctoral thesis from which this article emerged and reviewing more recent work on employee responses to air travel, as well as per your suggestions. Several edits were made to ensure it is clear to the reader that the article provides evidence to demonstrate, largely in line with previous literature, that awareness is insufficient; action must be taken. Some suggested actions which emerged from the interviews and research were added in a new paragraph in the conclusion.
- Methodology – in the opinion of the main author, piloting, NDA and ethical clearance are important in this case. Perhaps the name of the interviewer and alternative software could be removed.
- Results – this section also contains several edits. Clearer figure captions. A reduction in the repetition of the survey questions. An explanation of how the combined variables were created. A few quotes from the interviews were added, and the broader “inputs of value for a discussion of broader corporate culture” from the interviews, which was in the conclusion in the previous version, have been brought up in the results section. Concerning the H(5) justification for the usage of hypothetical questions within the set, it was explained in a new paragraph as part of the discussion of the quantitative methodology, with reference to appropriate literature. References and evidence have been added.
- All other comments have been partially implemented where possible.

In the hope that these edits will work to your satisfaction, I would greatly appreciate if you could review the article and its rating, once the new version has been published in the next couple of days. 

 Kind regards

 Ignacio Echeverria Arrondo

# Response

**Published:** 2022-04-24

**Authors:** Ignacio Echeverria Arrondo

**Affiliations:** SBS Swiss Business School , Switzerland

Dear reviewer,

 Thank you for your detailed feedback and please excuse the delay responding, as I was waiting for more reviews.

- Methodology – as for the sample size, the minimum of 400 respondents (above 384) was requested for the quantitative section of the dissertation from which this article has been extracted, to be accepted (Mugenda and Mugenda 2012). The number of valid survey participants was 104. A comment has been added in the results section; ‘while this sample was sufficient for this mixed-methods study, it is below the 384 required for the quantitative data to be taken as statistically representative of the population being studied. Future researchers may want to replicate this study to a larger sample, and also to take account of the advice in van Mol (2015) and provide repeated digital reminders for potential participants. To prevent lengthening the quantitative survey, which might have further impacted the response rate, demographic data was not collected’.
- Methodology –  the demographic estimated data has been removed as suggested.
- Background literature – more sources have been added to back up the hypotheses.
- Results – survey questions have been removed as suggested.
- Abstract – the results section has been amended although the precise results are still included. In the conclusion ‘research for this article was undertaken prior to COVID-19, so future research will be necessary to investigate the new context’ has been added.
- Introduction – ‘corona disease 2019 pandemic’ has been rephrased. Also, in-text citation has been amended.
- Literature background – Tani’s first name initial has been removed, as suggested.
- In the objective section, the word ‘simple’ has been removed, as suggested.
- In the opinion of the main author, the questionnaire tests, the NDA and ethical approval are important. Perhaps the name of the interviewer and alternative software could be removed as suggested.
- The results of the interviews have been moved from the discussion to the results section.
- All other comments have been partially implemented where and if possible. 

In the hope that these edits will work to your satisfaction, I would greatly appreciate if you and/or your co-reviewers could review the article and its rating, once the new version has been published in the next couple of days. 

 Kind regards

 Ignacio Echeverria Arrondo
